# Visual Performance Fields: Frames of Reference

**DOI:** 10.1371/journal.pone.0024470

**Published:** 2011-09-08

**Authors:** Jennifer E. Corbett, Marisa Carrasco

**Affiliations:** Departments of Psychology and Center for Neural Science, New York University, New York, New York, United States of America; Kyushu University, Japan

## Abstract

Performance in most visual discrimination tasks is better along the horizontal than the vertical meridian (*Horizontal-Vertical Anisotropy, HVA*), and along the lower than the upper vertical meridian (*Vertical Meridian Asymmetry, VMA*), with intermediate performance at intercardinal locations. As these inhomogeneities are prevalent throughout visual tasks, it is important to understand the perceptual consequences of dissociating spatial reference frames. In all studies of performance fields so far, allocentric environmental references and egocentric observer reference frames were aligned. Here we quantified the effects of manipulating head-centric and retinotopic coordinates on the shape of visual performance fields. When observers viewed briefly presented radial arrays of Gabors and discriminated the tilt of a target relative to homogeneously oriented distractors, performance fields shifted with head tilt (Experiment 1), and fixation (Experiment 2). These results show that performance fields shift in-line with egocentric referents, corresponding to the retinal location of the stimulus.

## Introduction

**Figure 1:**
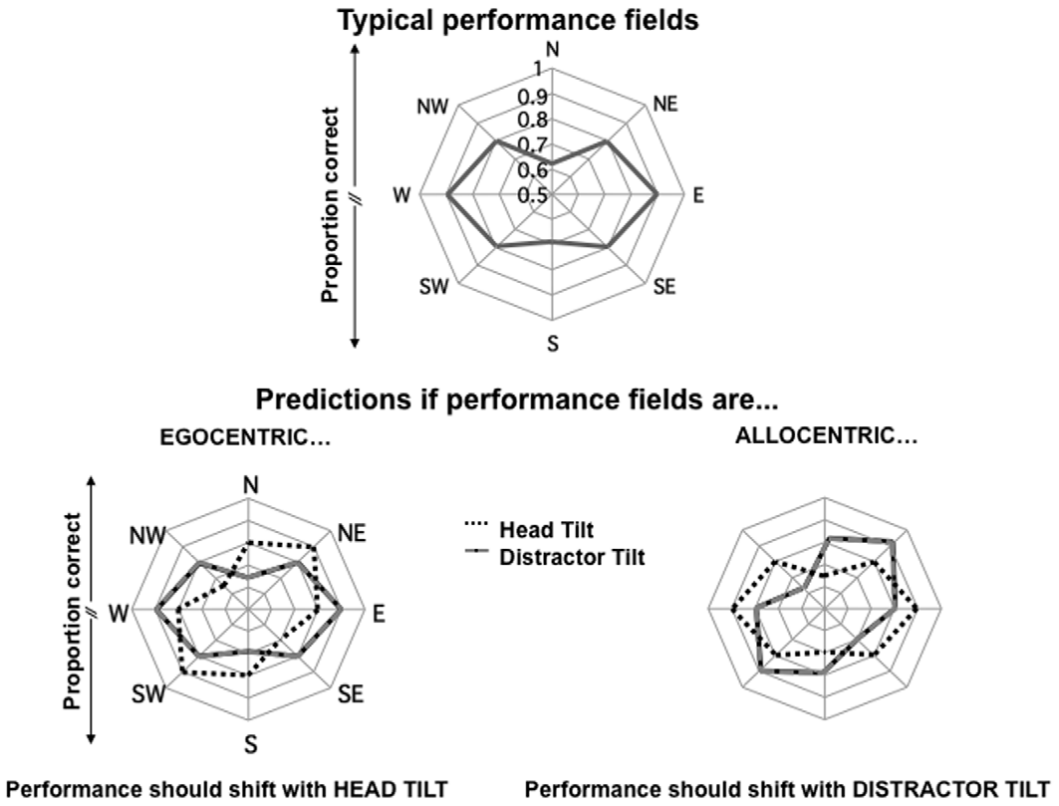
*Top*: A typical observer's performance fields (center = 0.5, chance performance) with an apparent: 1) Horizontal-Vertical Anisotropy (HVA): better performance at isoeccentric locations along the horizontal (East–E and West–W locations) than vertical (North–N and South–S locations) meridian of the visual field, and 2) Vertical Meridian Asymmetry (VMA), better performance in the location directly below fixation (S) than directly above (N). *Bottom*: Hypothesized performance based on egocentric coordinates (tilting the observer's head should result in a corresponding shift in the associated performance fields), versus an allocentric frame of reference (tilting the stimuli should shift performance fields).

Discriminability and the speed of information processing differ as a function of eccentricity and isoeccentric locations across the visual field [1]–[5]. Formally, the term *performance field* is used to describe the fact that performance is not homogeneous at isoeccentric locations [6]–[7]. Figure 1 (top) depicts typical performance fields across isoeccentric cardinal and intercardinal locations, showing a *Horizontal-Vertical Anisotropy (HVA)*: better performance on the horizontal (East–E and West–W locations) than vertical (North–N and South–S locations) meridian of the visual field, and a *Vertical Meridian Asymmetry (VMA)*: better performance in the location directly below fixation (S) than directly above (N), with intermediate performance at intercardinal locations (NE, NW, SE & SW) [2], [5], [8]. In general, their characteristic shape reflects the canonical layout of salient stimuli in the external environment (e.g., the majority of important visual events occur along or below the horizon, and we rarely monitor the location directly above fixation).

Performance fields are pervasive in vision. They affect a wide variety of detection, discrimination, and localization tasks mediated by contrast sensitivity and spatial resolution [1],[2],[5],[7]–[24]. Performance fields show a characteristic shape for different stimulus orientations and luminance levels, with both monocular and binocular viewing conditions [5]. These asymmetries become more pronounced as eccentricity, spatial frequency, and the number of distracters are increased [2], [5], [11], [15]. Yet, in all studies to date, their overall shape remains significantly reliable, with only minor fluctuations over different observers [2], [5], [8], [25]. However, in all of these previous investigations, observer-centered and environmental references have been aligned. Given the numerous situations in which these reference frames can become dissociated (e.g., when an observer's head is tilted, or when gazing sideways), here we tested how performance fields translate when these egocentric and allocentric coordinates are decoupled. Before describing our specific experiments, we first review the relevant dissociations between egocentric and allocentric reference frames, and how such manipulations affect related visual phenomena.

### Egocentric and allocentric spatial reference frames

Despite the subjective impression that the brain constructs a unitary spatial map of the world, we reference multiple contexts depending on the task at hand. These reference frames can be divided into two main categories: egocentric, observer-centered coordinates, and allocentric, environment-centered coordinates [26]–[30]. Egocentric reference frames can be retinotopic, head- or trunk-centered, or hand–shoulder-centered. Allocentric reference frames can be based on the direction of the pull of gravity (geocentric frame), or on the visual context of the surrounding environment (pattern-centric frame). Numerous experimental findings reflect dissociations between egocentric and allocentric spatial reference frames. For example, an fMRI study revealed that only a subset of regions in the bilateral fronto-parietal network involved in egocentric processing were active during object-based, allocentric processing [31]. In addition, patients with parietal damage resulting in unilateral visual neglect can be unaware of the opposite side of the body, or neglect the contralateral side of objects or the surrounding visual environment [32]–[36].

### Perceptual consequences of spatial reference frame dissociations

Egocentric and allocentric manipulations differentially bias a range of visual phenomena. Many aspects of vision are modulated by both types of reference frames. For example, the visual *Class 1 oblique effect* (our superior ability to process cardinally oriented stimuli versus oblique stimuli), is specified in terms of purely retinal coordinates, but the more cognitive, *Class 2 oblique effect* (our superior memory for cardinal versus oblique orientations) is also affected by allocentric inputs, including proprioceptive information regarding the orientation of supporting surfaces and the orientation of the observer relative to the pull of gravity [37], [38]. Similarly, the lower region cue to figure-ground segregation (our tendency to perceive lower regions as figures; [39]) is based on environmental depth considerations, such that the side of the display attached to the receding depth plane in the terrestrial gravitational environment is most often perceived as “figure” [40]. Yet, segregation is also governed by an egocentric frame of reference, such that this figure/ground bias translates along with head tilt [41].

Other visual phenomena, such as our superior ability to discriminate right angles with vertical and horizontal sides versus right angles with oblique sides (the Goldmeier effect; [42]), are mainly affected by an allocentric frame of reference; when the head is tilted 45^o^ from gravitational vertical, the gravitationally normal right angle is still perceived better than a gravitationally oblique (45°) angle aligned with the orientation of the head [43], [44]. On the other hand, the *Central Performance Drop (CPD)* (superior texture segmentation in parafoveal versus foveal locations; [45]–[47]) is driven by purely egocentric referents, in particular, the disproportionate representation of the central ∼2° of the visual field throughout the visual system.

As performance fields are prevalent throughout visual tasks, it is important to understand their perceptual consequences. For instance, differences in the speed of temporal processing may account for lower signal-to-noise thresholds for detecting coherently moving dot patterns along the horizontal versus vertical meridian [48], and may underlie the fact that observers are more prone to the line motion illusion at isoeccentric locations along the upper than the lower vertical meridian [49]. Considering the myriad of critical situations when an observer must quickly and accurately process visual information from an angle, or out of the corner of one eye (e.g. driving with the head forward while monitoring a navigational device situated off to the side), it is particularly important to determine how performance fields are manifest when egocentric reference frames are decoupled from the canonical allocentric reference frame given by the layout of the surrounding environment and the pull of terrestrial gravity. Towards this end, we conducted the present study to examine how performance fields shift with head tilt (Experiment 1) and fixation (Experiment 2).

## Experiment 1: Head Tilt

To assess the contributions of egocentric and allocentric reference frames, we measured observers' performance fields under four different circumstances: 1) when their heads were upright and they viewed upright displays, 2) when their heads were upright and they viewed displays of tilted stimuli, 3) when their heads were tilted and they viewed upright stimuli, and 4) when their heads were tilted and they viewed tilted stimuli. If performance fields are affected by changes in egocentric frames of reference, tilting the observer's head should result in a corresponding shift in performance. If performance fields are affected by changes in allocentric frames of reference, tilting the stimuli should similarly affect performance. Figure 1 (bottom) illustrates the results expected for each type of effect. As a final consideration, if performance fields are differentially mediated by both egocentric and allocentric frames of reference, these effects should interact when both the head and display are tilted congruently.

### Methods

#### Observers

Four observers (all male, aged 23–50 years old), all with normal or corrected-to-normal vision participated in two, one-hour long experimental sessions; one with their heads upright and one with their heads tilted. All participants gave written informed consent prior to the start of the experiment. New York University's Institutional Review Board approved all procedures and protocols.

#### Apparatus

Matlab and the Psychophysics Toolbox [50], [51] were used to control all the display, timing, and response functions. Observers viewed the stimuli on a gamma-corrected monitor [52]. A video attenuator was used to drive only the green gun of a 53 cm (diagonal) IBM P260 monitor (1024×768; 120 Hz). Background luminance was set to the middle of the monitor's range (16 cd/m^2^). To minimize the contributions of the upright context of the monitor and surrounding testing room, we affixed a black cardboard annulus with an outer diameter of 45 cm and an inner diameter of 32 cm around the center of the computer monitor, and kept the room's lights off for the entire duration of each experimental session.

In the upright head session, we secured participants' heads in a vertical (0°) position using a traditional combination chin-and-head rest. In the tilted head session, we secured their heads at a −45° counterclockwise (CCW) (left) tilt from vertical about an imaginary x-axis passing through the center of each eye using a custom-made padded chin-and-head rest. This head tilt manipulation caused observers' heads to be rotated −45°CCW around the central fixation point.

#### Stimuli

**Figure 2:**
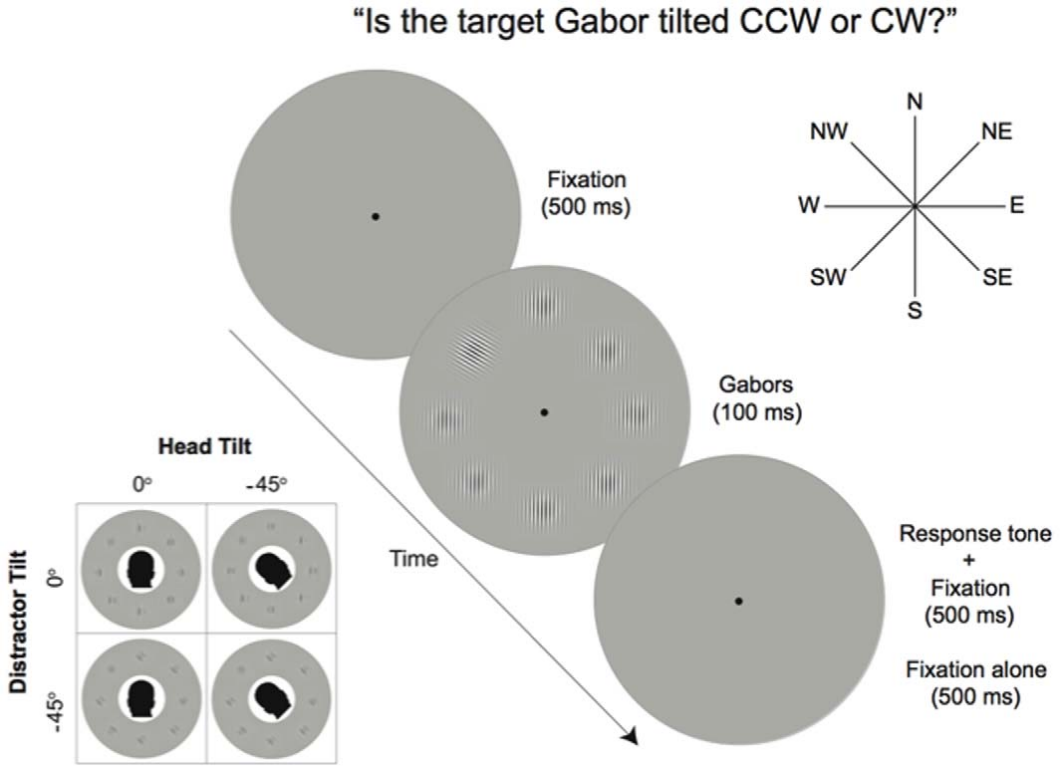
Observers viewed briefly presented radial arrays of eight suprathreshold Gabors at four cardinal and four 45° intercardinal locations, equidistant from fixation, and determined the CW versus CCW tilt of a target Gabor (in this example, the target is tilted CCW in the NW position). Each trial began with the fixation dot presented alone in the center of the display for 1000 ms. Next, the stimulus display of eight Gabors was also presented centered around the fixation dot for 100 ms, followed by a 400 Hz tone response prompt and the fixation dot for 500 ms, and then only the fixation dot for another 500 ms. To dissociate egocentric and allocentric coordinates, each participant viewed vertical (0°) distractors with CW and CCW tilted targets (±60°) and −45° CCW tilted distractors with tilted targets (−105°, +15°) in both upright (0°) and −45° CCW tilted head postures.

Observers viewed briefly presented radial arrays of eight suprathreshold Gabors at four cardinal and four 45° intercardinal locations relative to the center of the circular viewing window. The Gabors were presented at eight equidistant locations (at the cardinal and intercardinal locations) from a central fixation point on an invisible polar grid at 6° of eccentricity (Figure 2). Each Gabor patch subtended 2° of visual angle, on the basis of a fixed 114 cm viewing distance, and had a center spatial frequency of 6 cpd.

#### Procedure

Each observer participated in two sessions on separate days; one with the head upright, and one with the head tilted. The order of the sessions was counterbalanced across observers. In each session, they determined whether a target tilted relative to the homogeneously oriented distractor Gabors was tilted clockwise (CW) (right), or counterclockwise (CCW) (left) on each trial. We varied the orientation of the distractors in both experimental sessions, such that each participant viewed vertical (0°) distractors with CW and CCW tilted targets (±60°), and −45° CCW tilted distractors with tilted targets (−105°, +15°), in both upright (0°) and −45° CCW tilted head postures (as illustrated in Figure 2 ). To allow for a measure of any interaction between ego- and allocentric effects, we always tilted the head and stimulus display in the same (−45° CCW, leftward) direction. Although there was no a priori reason to suspect that performance would differ between leftward and rightward head tilts, we conducted a pilot experiment in which observers' heads were positioned in three orientations: 1) −45° leftward, 2) 45° rightward, and 3) 0° upright in each of the three corresponding distractor tilt conditions: 1) −45° leftward, 2) 45° rightward, and 3) 0° upright. Given that performance fields always shifted with head tilt, and never with distractor tilt, we did not conduct the full factorial design in the main experiment.

The location of the tilted target varied randomly among the eight possible cardinal and intercardinal points. An adaptive staircase procedure, QUEST [53], was used to determine each observer's contrast threshold for the Gabor stimuli to perform the task with 75% accuracy across all locations in each of the four experimental conditions (2 Head Postures * 2 Distractor Orientations).

Figure 2 illustrates a sample trial sequence. A fixation dot subtending 0.2° of visual angle remained visible in the center of the display throughout the experiment. Each trial began with the fixation dot presented alone in the center of the display for 1000 ms. Next, the stimulus display of eight Gabors was also presented, centered around the fixation dot for 100 ms, followed by a 400 Hz tone response prompt and the fixation dot for 500 ms, and then only the fixation dot for another 500 ms.

In each of the two sessions (Head Upright, Head Tilted), the target appeared in one of the eight possible locations at random, 14 times per block, resulting in 112 trials in each of the eight possible target locations. To maximize comfort, observers were given a short break after each block of trials so they could straighten their heads and stretch their necks to sooth any discomfort induced in the tilted head condition.

### Results

**Figure 3:**
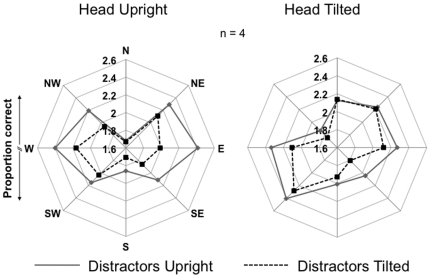
Experiment 1 results: Performance fields shifted with the position of the head, not the distractors. Observers' (n = 4) average performance (in 2arcsin(sqrt(accuracy)) units) for upright (graph on left) and tilted (graph on right) head postures, and upright (solid lines) and tilted (dashed lines) distractors at each of the 8 possible target locations. Note that the SEMs are not shown, as they were too small across all data points to be visually useful.

Overall, performance fields shifted with the position of the head (Figure 3). We obtained each observer's average accuracy and SEM for each combination of Head Posture, Distractor Orientation, and Target Location. Note that the error estimates for each location were too small to be visually useful in Figure 3 (average *SEM* = .02). We next took the 2arcsin(sqrt(x)) transform of each of these individual accuracy values to minimize distortion in the data due to restricting the range of possible performance to an upper limit of 1.0 (100%) [54], and applied this transformation to all subsequent data in this study. As per previous investigations of visual performance fields [5], [8], we first conducted a 2 (Head Posture) * 2 (Distractor Orientation) * 8 (Target Location) omnibus ANOVA on these transformed values averaged over participants, which confirmed main effects of Head Posture (*F*(1,3)  = 13.474, *MSE* = .139, *p* = .035, *η^2^* = .818), and Location (*F*(7,21)  = 11.521, *MSE* = .427, *p*<.001, *η^2^* = .793), but only a marginal trend for the main effect of Distractor Orientation (*F*(1,3)  = 6.104, *MSE*  = .841, *p* = .09, *η^2^* = .670). There were also significant interactions between Head Posture and Location (*F*(7,21)  = 8.214, *MSE* = .197, *p*<.001, *η^2^* = .732), and Distractor Orientation and Location (*F*(7,21)  = 4.091, *MSE* = .035, *p* = .006, *η^2^* = .577), but no interaction between Head Posture and Distractor Orientation (*F*(1,3) = .548, *MSE* = .059, *p* = .513, *η^2^* = .154).

Figure 3 clearly illustrates that performance fields shifted with head tilt, regardless of the orientation of the distractors. Individual observers' performance fields also followed this pattern. Therefore, we collapsed the data across the two types of distractors (0° and −45°) to further compare performance fields when the head was upright versus tilted. There was a significant HVA, such that performance was superior along the horizontal meridian (W & E locations) than the vertical meridian (N & S locations) when observers' heads were upright (*t*(7)  = 9.317, *SEM* = .054, *p*<.001), and along the horizontal meridian with respect to head tilt (SW & NE locations) than the vertical meridian with respect to head tilt (NW & SE locations) when their heads were tilted (*t*(7) = 6.380, *SEM* = .065, *p*<.001). Although Figure 3 illustrates the tendency for superior performance in the S versus N locations when observers' heads were upright, and in the SE versus NW locations when their heads were tilted, this VMA was not significant in either condition. Performance at intercardinal locations (NW, NE, SE, & SW) was intermediate between performance along the horizontal (E & W) (*t*(7)  = 1.884, *SEM* = .07, *p* = .102), and vertical (N & S) meridians (*t*(7)  = −4.641, *SEM* = .079, *p* = .002) when observers' heads were upright, and performance at intercardinal locations with respect to head tilt (N, S, E, & W) was intermediate between performance along the horizontal (SW & NE) (*t*(7)  = 5.205, *SEM* = .046, *p* = .001) and vertical (NW & SE) meridians (*t*(7)  = −2.765. *SEM* = .063, *p* = .028) when their heads were tilted.

### Discussion

The results of Experiment 1 support the proposal that performance fields shift with head tilt and are specified in terms of egocentric coordinates. Overall, observers exhibited characteristic performance asymmetries when the head was upright, which shifted with the tilt of the head, independently of the orientation of the distractors. This conclusion is supported by a main effect of Head Posture, but no main effect of Distractor Orientation and no interaction between Head Posture and Distractor Orientation on observers' transformed average accuracy. Coupled with Figure 3, the observed interaction between Head Posture and Target Location likely reflects an egocentric shift in performance fields with Head Posture. On the other hand, the interaction between Distractor Orientation and Target Location probably reflects lower overall accuracy at most locations when the distractors were tilted, perhaps due to the visual oblique effect [37], [38].

There was evidence of a strong HVA, as given by the significant differences between performance at isoeccentric locations along the horizontal and vertical meridians in both the Head Upright and Head Tilted conditions. The VMA was weak in comparison to this HVA; yet, performance in the S location tended to be superior to performance in the N location in the Head Upright condition, and in the SE versus NW locations in the Head Tilted condition. Performance was intermediate at the intercardinal locations relative to performance at isoeccentric locations along the horizontal and vertical meridians (Figure 3). Importantly, the lack of a statistically significant VMA does not detract from the study's main goal to determine whether performance fields shift as a function of stimuli relative to an egocentric frame of reference. The crucial result in Experiment 1 is that, overall, the pattern of performance when observers' heads were upright shifted in-line with head tilt.

The results of Experiment 1 provided strong evidence that performance fields are linked to an egocentric versus allocentric frame of reference. However, in all conditions, the position of the head was aligned with a central fixation dot. Under these circumstances, it is impossible to parse retinotopic from head-centric contributions to the observed inhomogeneities in performance. Therefore, in Experiment 2, we chose to further dissociate these two egocentric reference frames by fixing observers' heads in a constant upright position aligned with the center of the monitor while shifting the location of fixation.

## Experiment 2: Fixation Shifts

Whereas the results of Experiment 1 demonstrated that performance fields shifted with the tilt of the head and not with the tilt of the distractors, these findings did not allow us to decouple two specific types of egocentric references: retinotopic and head-centric coordinates. Therefore, in Experiment 2, we dissociated these reference frames by fixing observers' heads in a constant upright posture and varying the location of fixation relative to radial arrays of Gabors arranged at eight equidistant locations about the center of the circular viewing window aligned with the center of the head. It is well established that performance declines as a function of eccentricity proportional to cortical representation [55]. In addition to accounting for this systematic decline, here we specifically tested whether the characteristic asymmetries of performance fields would translate in the fixation shift condition to match performance fields modeled using stimuli presented at corresponding head-centric and retinotopic coordinates. If performance fields are retinotopic, they should shift as a function of the location of the individual targets relative to the location of the shifted fixation dot. However, if performance fields are mediated by the position of the head, then no shift should be observed, and performance fields should be similar to those obtained using a central fixation dot and centered displays, as in Experiment 1.

### Methods

#### Observers

Four observers (3 NYU graduate students and 1 postdoctoral fellow; 1 female; aged 21–30 years old) participated in two, one-hour long experimental sessions, all with normal or corrected-to-normal vision. One of these observers also participated in Experiment 1. All participants gave written informed consent prior to the start of the experiment. New York University's Institutional Review Board approved all procedures and protocols.

#### Apparatus

The basic set-up was as in Experiment 1, except that we restrained the head in a constant vertical position while shifting the location of the fixation dot to the N, S, E, and W of the center of the display and the head. To ensure a fixed upright posture for the duration of each experimental session, we positioned observers in the standard combination chin-and-headrest 114 cm in front of the computer monitor, and added foam padding until the head was held firmly, but comfortably in place, and the center of the eyes was aligned with the center of the computer monitor and circular viewing window. In addition, to ensure that observers were fixating as directed, we tracked the position of the center of the right pupil on each trial using an Eyelink 1000 connected to a Macintosh G4 computer. In Experiment 2, stimuli were presented on a 53 cm (diagonal) Dell monitor (1024 · 768; 120 Hz), without the video attenuator used in Experiment 1. We excluded from analysis any trials in which eye position shifted more than 1° in any direction from the center of the particular location of the fixation dot. As observers were all highly experienced in psychophysical experiments using eye-trackers, this criterion excluded <1% of trials per condition, per observer.

#### Procedure

To dissociate the position of the image on the retina from the position of the image relative to the head, we presented 2cpd Gabors subtending 2° of visual angle at eight equidistant cardinal and intercardinal locations, 6° of eccentricity from the center of the circular viewing window (as in Experiment 1), but varied the location of a fixation dot subtending 0.2° of visual angle by 4° randomly to the N, S, E, or W of the center of the display and the head. We then measured each observer's orientation discrimination accuracy at each of the eight possible target locations for N, S, E, and W fixation shifts. Observers performed four blocks of 400 trials, yielding 50 trials of each of the 32 possible combinations of 4 Fixation Shifts (N, S, E, and W), and 8 Target Locations (N, NE, E, SE, S, SW, W, and NW).

As a result of these Fixation Shifts, the Gabors in each experimental display were equidistantly arranged around the center of the display and the center of the head, but no longer equidistantly arranged around fixation. Instead, individual Gabors in each display could be differentially displaced 2°, 4.25°, 7.21°, 9.27°, or 10° of visual angle to the N, S, E, or W of the center of the shifted fixation dot. Table 1 lists the specific displacements at each of the eight possible target locations relative to a fixation dot shifted 4° of visual angle to the N, S, E, and W of the center of the head/monitor.

**Table 1:** Experiment 2, Fixation Shifts.

*Target Location*								
*Fixation Shifts*	N	NE	E	SE	S	SW	W	NW
**East**	7.21	4.25	2	4.25	7.21	9.27	10	9.27
**West**	7.21	9.27	10	9.27	7.21	4.25	2	4.25
**North**	2	4.25	7.21	9.27	10	9.27	7.21	4.25
**South**	10	9.27	7.21	4.25	2	4.25	7.21	9.27

Eccentricities (in degrees of visual angle) of individual target locations as a function of *retinotopic* coordinates, relative to a fixation dot shifted 4° to the N, S, E, and W of the center of the head and monitor.

To test our hypothesis that the location of individual stimuli relative to fixation was the key variable affecting the shape of observers' visual performance fields, we next measured each participant's performance fields using displays of Gabors again arranged at eight equidistant cardinal and intercardinal locations around a central fixation point (as in Experiment 1). We then used these measurements to predict performance both as a function of each target's *retinotopic* location relative to fixation, and as a function of each target's *head-centric* location:

#### Retinotopic predictions

To test whether performance fields shifted relative to retinotopic coordinates, we compared average 2arcsin(sqrt)-transformed performance in the trials with shifted fixation dots to average 2arcsin(sqrt)-transformed performance for trials with targets offset at corresponding locations from a central fixation dot. Specifically, we measured accuracy at each of the 32 locations listed in Table 1 relative to a central fixation dot using five different rings of eight Gabors, arranged isoeccentrically at five different distances from a central fixation dot: 2°, 4.25°, 7.21°, 9.27°, and 10°. We then used each observer's performance in these trials with targets isoeccentrically displaced relative to a central fixation dot to model expected performance for trials in which targets were presented at corresponding retinal displacements relative to a shifted fixation dot. For example, for trials in which the fixation dot shifted 4° to the East, targets in the West displaced 6° from a central fixation dot, became displaced by 10° from the East-shifted dot. Were performance fields a function of the *retinotopic* locations of individual targets, performance for these trials should be similar to performance for trials in which targets were displaced by the corresponding *retinotopic* distance of 10° to the West of a central fixation dot.

#### Head-centric predictions

To predict each observer's performance fields based on head-centric coordinates, we used the 2arcsin(sqrt) transform of individual performance measured at each possible target location in trials with Gabors arranged isoeccentrically 6° around a central fixation dot, the center of the head, and the center of the monitor, exactly like in Experiment 1. Were performance fields a function of the *head-centric* locations of individual targets, performance for these trials should be similar to performance for trials in which targets were displaced by the corresponding *head-centric* distance of 6° to the West of a central fixation dot.

Observers performed one block of 400 trials with Gabors arranged around a central fixation at each of these six eccentricities (2°, 4.25°, 7.21°, 9.27°, and 10° to test *retinotopic* predictions, and 6° to test *head-centric* predictions), for a total of 2,400 trials; 50 trials for each of the eight possible target locations at each of the six eccentricities. We counterbalanced the order of the eccentricities over observers.

### Results

For each of the cardinal directions of Fixation Shifts (N, S, E, and W), we compared the average performance of each of the four observers at each of the corresponding eight target locations to their average performance for targets displaced from: 1) the center of the fixation dot (*retinotopic baselines*), and 2) the center of the head (*head-centric baselines*), respectively, by corresponding distances (listed in Table 1 ). Because fixation was neither aligned with the center of the monitor, nor with the center of observers' upright heads in Experiment 2, the Gabors were no longer equidistant from fixation. Therefore, we did not expect fixation shifts to preserve the canonical shape of performance fields that allowed for the straightforward analysis as in Experiment 1. Instead, we modeled the underlying distribution of performance expected as a function of retinotopic and head-centric target locations by bootstrapping performance in each of the 32 combinations of eight target locations and four (N, S, E, and W) fixation shifts, and compared this to bootstrapped performance at each of the 32 corresponding *retinotopic* and *head-centric* displacements listed in Table 1. This was done in Matlab in 10,000 iterations, each time sampling 10 responses from each of the 96 types of trials in Experiment 2 (with replacement after each iteration) for each observer (the 32 combinations of 4 Fixation Shifts and 8 Target Locations, the 32 corresponding retinotopic trial types, and the 32 corresponding head-centric trial types). On each iteration, the 2arcsin(sqrt)-transformed average accuracy of the 10 samples for each of the 96 data points was calculated. After iteration, the resultant 10,000 values for each of the 96 data points were averaged over observers.

Across the 4 observers, performance in the 32 combinations of 4 Fixation Shifts and 8 Target Locations was not significantly different from performance modeled at corresponding *retinotopic* locations, (*t*(127)  = .489, *SEM* = .01326, *p* = .626), but was significantly different from performance in corresponding *head-centric* locations (*t*(127) = 3.347, *SEM* = .01825, *p*<.001). This analysis indicates that performance was well predicted from retinotopic coordinates, but not from head-centric coordinates.

**Figure 4:**
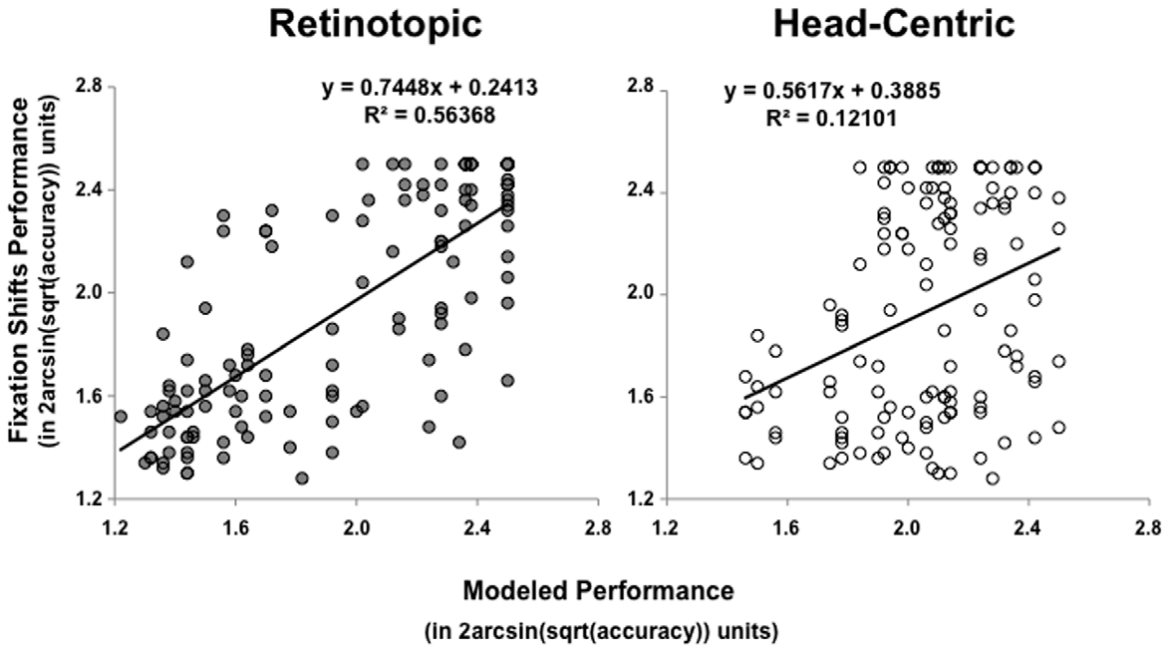
In Experiment 2, performance in the Fixation Shifts condition was well-predicted by performance in corresponding target locations with respect to *retinotopic* versus *head-centric* reference frames.

Furthermore, as illustrated by the scatterplots in Figure 4, over half of the variance in performance for trials in which fixation shifted relative to the center of the head/monitor was explained by performance modeled from trials in which targets were displaced to corresponding degrees from a central fixation (*R* ^2^ = .564; *F*(1,126)  = 162.78, *MSE* = 3.204, *p*<.001), In contrast, only 12% of the variance in performance for trials in which fixation shifted could be explained by performance modeled from trials in which targets were displaced to corresponding degrees from the center of the head (*R* ^2^ = .121; *F*(1,126)  = 17.35, *MSE* = .688, *p*<.001).

### Discussion

The results of Experiment 2 support the proposal that performance fields are *retinotopic* versus *head-centric* in nature. When fixation shifted, performance fields shifted according to *retinotopic* coordinates, to closely match performance modeled from stimuli presented at corresponding locations relative to a central fixation dot, but not according to performance modeled from stimuli presented 6° isoeccentrically around a central fixation dot corresponding to a head-centered origin. Importantly, the *head-centric* model predicts that the shape of performance fields should not change significantly as a function of the location of fixation, but remain approximately consistent with performance obtained using the 6° isoeccentric displays. Instead, performance fields shifted *retinotopically*, as a function of the individual target locations from fixation, as the *retinotopic* model accounted for more than four times as much of the variance in the observed performance compared to that accounted for by the *head-centric* model (Figure 4 ).

## Discussion

The present findings provide the first psychophysical evidence indicating that performance fields are mediated by a retinotopic frame of reference. In Experiment 1, although retinotopic, head-centric, and allocentric coordinates are normally aligned, when we dissociated these references, the shape of visual performance fields shifted with respect to the position of the observer's head, not the orientation of the distractors. In Experiment 2, when we further dissociated retinotopic and head-centered reference frames by restraining observers' heads in an upright posture, and varying the location of fixation dot relative to centered stimulus displays and the center of the head, performance shifted in close accordance with a retinotopically-based model, but was not well accounted for by a head-centric model. In all cases, performance fields shifted in-line with egocentric, retinotopic coordinates.

### Possible anatomical correlates of performance fields

Findings from several anatomical and physiological studies of macaque monkeys and humans suggest some possible physical correlates of visual performance fields. However, even combined, these findings cannot fully explain performance fields. The lower density of ganglion cells and faster decline in cone density over increasing distances from the fovea along the vertical versus horizontal meridian [56]–[59] are most likely correlates of the HVA. There is also evidence for a similar HVA in the LGN [60] and V1 [61], [62].

The anatomical correlates of the VMA are much less clear. The higher density of magno-cells (primarily responsible for temporal processing) on the corresponding regions of the retina [63] may explain why there is slightly more area devoted to the inferior than superior visual field in the LGN [60] and V1 [61], [62], and why the maps of the visual field in MT [64] and lateral occipital (LO) cortex are biased toward the lower visual field [65]. In addition, the fibers in the Meyer's Loop, or anterior extension of the geniculocalcerine tract carrying information from the upper visual field from the LGN to the occipital cortex travel a slightly longer route around the temporal horn than the posterior fibers carrying information about the lower visual field. Also, there is less direct input from layer 4B in V1 to the upper than the lower map in V3/VP [63]. Note, however, that although such differences may be correlates for the VMA, no physiological or anatomical asymmetries between the upper versus lower visual fields have been specifically localized to the vertical meridian. Unfortunately, the vertical meridian is scarcely recorded electrophysiologically because it is near the boundaries between visual areas [66]. Interestingly, human fMRI results reflect retinotopic neural correlates of the VMA in V1 and V2. However, the asymmetric BOLD activity at the upper and lower regions of the vertical meridian could be due to neuronal density, extent of activation, or both [15].

Although current anatomical and physiological knowledge cannot fully explain the canonical shape of visual performance fields with respect to the VMA and performance at intercardinal locations, the present findings are consistent with the possible anatomical and physiological correlates of performance fields outlined above [15], [56]–[64]. To further speculate, the slightly greater representation in macaque V1 of the inferior than superior visual field, and the substantially greater representation of the visual field ±45° around the horizontal meridian relative to the representation of the visual field ±45° around the vertical meridian reported by Van Essen [62] may reflect asymmetries specific to the region surrounding the vertical meridian, and could explain findings that performance at intercardinal locations is intermediate to performance along the vertical and horizontal meridians [2], [5], [8].

Furthermore, physiological asymmetries responsible for performance fields likely exist not only in the retina, but are also found throughout the hierarchy of visual information processing; in the LGN, V1, and possibly even extrastriate areas such as MT. Along these lines, Silva and colleagues [67], [68] report functional and structural asymmetries in correspondence with performance anisotropies observed in a detection task mediated by contrast sensitivity between the nasal and temporal regions of the visual field, which they attribute to retinal factors, and between the left and right visual fields, which they attribute to cortical factors. As targets were presented only in intercardinal locations, their findings cannot be extended to inform about the retinal or cortical nature of the visual performance fields under investigation in the present study, but do warrant further investigation concerning the physical nature of characteristic performance inhomogeneities across the visual field. Towards these ends, future studies exploiting the spatial and temporal benefits of fMRI and EEG measures may help to better isolate the differential retinal, and cortical contributions that underlie visual performance fields.

### Implications for visual displays

These varied effects of egocentric and allocentric manipulations on visual perception underscore the importance of understanding how spatial reference frames mediate visual performance fields. Characterizing how the typical shape of visual performance fields is affected when the position of the head, the environment, and the retinal image are dissociated will not only increase our understanding of how to design and interpret a range of vision studies, but will also allow for a better understanding of how critical information, such as instrument panels and warning signals could be presented to capitalize on these performance inhomogeneities.

Along these lines, the systematic inhomogeneities in performance across the visual field described here and in numerous other studies [1]–[25], [49], [69] are clearly a crucial consideration for the design and interpretation of a wide variety of visual detection, discrimination, and localization tasks. In fact, performance fields are so pronounced that a handful of studies have avoided presenting stimuli at the horizontal and vertical meridians, instead presenting them only near intercardinal locations when averaging performance across the visual field [70]–[73].

Yet, although vision research regularly accounts for similar constraints imposed by cortical magnification factors and visual context effects, performance is often assessed independently of the target's isoeccentric location in the visual field. In fact, several studies have analyzed responses averaged across the visual field, obscuring any processing inhomogeneities (e.g. [74]; see [5] for a discussion). For example, Levine and McAnany [75] report superior performance in the lower versus upper visual field for a variety of tasks mediated by luminance, color, motion in depth, relative disparity, and lateral motion. Although stimuli were presented in the N and S locations along the vertical meridian, as well as to the left and right of these cardinal locations, results were collapsed across each of the three locations in the upper and lower visual fields, making it impossible to discern whether performance asymmetries along the vertical meridian were otherwise responsible for their reported lower visual field advantages.

In addition to obscuring performance inhomogeneities by averaging over isoeccentric target locations, others have generalized results from the vertical meridian to the upper and lower visual fields. For instance, an examination of previous studies suggest that in the greater susceptibility for perceiving illusory contours in the lower versus upper visual field reported by Rubin, Nakayama, and Shapley [76] is, in fact, driven by differences in performance along the vertical meridian, the only locations that were tested [5], [24].

Besides being frequently discounted in the design of visual displays, performance fields have been repeatedly misattributed to attentional factors, without empirical confirmation. For example, the HVA observed in a letter identification task [7] and an upper versus lower visual field asymmetry observed in half of the participants in a Snellen acuity task [6] have been ascribed to effects of sustained attention. Likewise, He and colleagues [77] attributed the upper/lower visual field asymmetry reported in search tasks to higher attentional resolution in the lower visual field. However, these conclusions were discordant with findings from investigations of the effects of systematic manipulations of visual and attentional factors on the shape of visual performance fields, which revealed that their canonical shape was consistent across attentional and control conditions in orientation discrimination [5], [8], acuity [69], texture segmentation [24], and feature and conjunction search tasks [74], [78]–[80]. All these results demonstrated that the canonical shape of visual performance fields was indeed unaffected by visual attention, and that a more parsimonious explanation for the findings of Mackeben [7] and Altpeter and colleagues [6] outlined above was given by purely visual factors.

Our present findings raise another important ecological consideration for tasks that require high resolution: whether the visual system constrains performance for tasks mediated by contrast sensitivity and spatial resolution similarly across egocentric and allocentric coordinates. Along these lines, Rubin and colleagues [76] have proposed that the greater tendency to perceive illusory contours in the lower visual field may be the result of a superior survival strategy; scene segmentation is most salient along the ground plane in the lower visual field where the majority of important events occur. As both contrast sensitivity and spatial resolution underlie the processing of all visual stimuli, we have previously confirmed that both dimensions are, in fact, superior in isoeccentric S versus N locations along the vertical meridian, not just uniformly superior across the lower versus upper visual fields [2], [5], [8], [9], [15], [24], [81]. However, according to Previc [20], the upper/lower visual field asymmetry may be a result of functional specialization where the upper visual field processes distant information and the lower visual field processes more proximal information. Therefore, future research is necessary to determine whether tasks mediated by contrast sensitivity are governed more by allocentric coordinates because it is important for individuating objects at a distance, whereas tasks mediated by spatial resolution are linked to egocentric coordinates because it is needed to identify objects closer to the observer.

Finally, the present results clearly indicate that performance fields shift with respect to the location of stimuli on the retina. However, we cannot completely rule out some influence of the orientation of the allocentric environment. Future investigations manipulating the physical tilt of the experimental room or manipulating the physical tilt of the observer and/or the orientation of the surrounding context of the displays to more extreme extents than in the present investigation may shed light on any undiscovered allocentric and/or head-centric contributions to the performance fields. Importantly, here we do show that performance fields are largely associated with retinotopic coordinates in that both a significant HVA, and the typical pattern of intermediate performance at intercardinal locations versus performance at locations along the horizontal and vertical meridians shifted with head tilt.

In summary, our results provide the first psychophysical confirmation that performance fields are retinotopic. The retinotopic nature of performance fields has yet to be completely explained by findings from anatomy, physiology, or neuroimaging. In closing, we strongly caution that these pervasive inhomogeneities in processing across the visual field be routinely accounted for in the design and interpretation of subsequent studies, especially when considering the best methods with which to present observers with critical visual information.
